# Histology of colonic submucosal lesions reveals a high proportion of benign lesions that do not require R0 en bloc endoscopic resection

**DOI:** 10.1055/a-2641-5256

**Published:** 2025-07-24

**Authors:** Pierre Lafeuille, Renato Medas, Benjamin Hamel, Romain Legros, Sarah Leblanc, Maximilien Barret, Vincent Lepilliez, Juliette Leroux, Thimothee Wallenhorst, Dann Joseph Ouizeman, Clement Fortier Beaulieu, Hugo Uchima, Elena De Cristofaro, Yann Le Baleur, Antoine Debourdeau, Fabien Subtil, Tanguy Fenouil, Alexandru Lupu, Florian Rostain, Jérôme Rivory, Jeremie Jacques, João Santos-Antunes, Mathieu Pioche

**Affiliations:** 1 26900 Edouard Herriot Hospital, Gastroenterology and Endoscopy Unit, Hospices Civils de Lyon, Lyon, France; 2 285211 Gastroenterology, Centro Hospitalar Universitário de São João, Porto, Portugal; 3 444193 Department of Gastroenterology, Villefranche-sur-Saône Hospital, Villefranche-sur-Saone, France; 4 Gastrenterology, Centre Hospitalier Universitaire Limoges Dupuytren, Limoges, France; 5 89686 Gastroenterology, Hopital Prive Jean Mermoz, Lyon, France; 6 568752 Hopital Cochin Gastroenterologie et oncologie digestive chirurgie digestive hepatobiliaire et endocrinienne, Paris, France; 7 89686 Gastroenterology, Hôpital Privé Jean Mermoz, Lyon, France; 8 36684 Department of Endoscopy and Gastroenterology, University Hospital Centre Rennes, Rennes, France; 9 37114 Gastroenterology Department, Centre Hospitalier Universitaire de Nice Hôpital L’Archet, Nice, France; 10 Gastroenterology Department, Kantys Clinique Saint George, Nice, France; 11 257333 Gastroenterology Department, Clinique de la Sauvegarde, Lyon, France; 12 16514 Endoscopy Unit, Hospital Universitari Germans Trias i Pujol, Badalona, Spain; 13 16711 Endoscopy Unit, Centro Médico Teknon, Barcelona, Spain; 14 60259 Gastroenterology, University of Rome Tor Vergata Faculty of Medicine and Surgery, Rome, Italy; 15 55662 Gastroenterology Unit, Fondation Hopital Saint Joseph, Paris, France; 16 26905 Gastroenterology Department, CHU de Montpellier, Montpellier, France; 17 26900 Biostatistiques, Centre Hospitalier Universitaire de Lyon, Villeurbanne, France; 18 36609 Service d'Anatomopathologie, Groupement Hospitalier Edouard Herriot, Lyon, France; 19 Service d'Hépato-gastro-entérologie, CHU Dupuytren Limoges, Limoges, France; 20 Gastrenterology, Centro Hospitalar S. João, Porto, Portugal; 21 70918 Glycobiology and Cancer, IPATIMUP, Porto, Portugal

**Keywords:** Endoscopy Lower GI Tract, Polyps / adenomas / ..., Tissue diagnosis, Endoscopic resection (polypectomy, ESD, EMRc, ...)

## Abstract

**Background and study aims:**

Submucosal lesions in the colon are much rarer than those in the rectum. Their nature is poorly understood, as is the best technique for their excision. Based on that of rectal lesions, it most often aims for R0 en bloc resection, but without formal proof of efficacy. The aim of this study was to evaluate histology of these lesions and determine whether submucosal lesions of the colon always require R0 en bloc endoscopic resection.

**Patients and methods:**

We conducted a retrospective international study of all colonic submucosal lesions with confirmed histology by resection or biopsy. We assessed the proportion of lesions correctly managed by endoscopy, so that the proposed resection technique offered a level of tumor resection quality appropriate to the definitive histology of the lesion.

**Results:**

One hundred patients with 105 colonic submucosal lesions from 13 European centers were included. Histology revealed 91.4% (96/105) non-malignant lesions and 8.6% (9/105) malignant lesions. Endoscopic techniques used were curative in 41.7% (5/12) of cases requiring resection, non-curative in 58.3% (7/12), and endoscopic resection was not necessary in 88.7% (93/105). There was no delayed surgery for adverse events.

**Conclusions:**

Most colonic submucosal lesions are non-malignant and do not warrant advanced endoscopic resection. A new therapeutic approach could be first-line use of a low-risk, low-cost histological diagnostic technique followed in a second phase by a more advanced technique in the event of a malignant histological result. Further studies are needed to evaluate this step-up strategy.

## Introduction

**Fig. 1:**
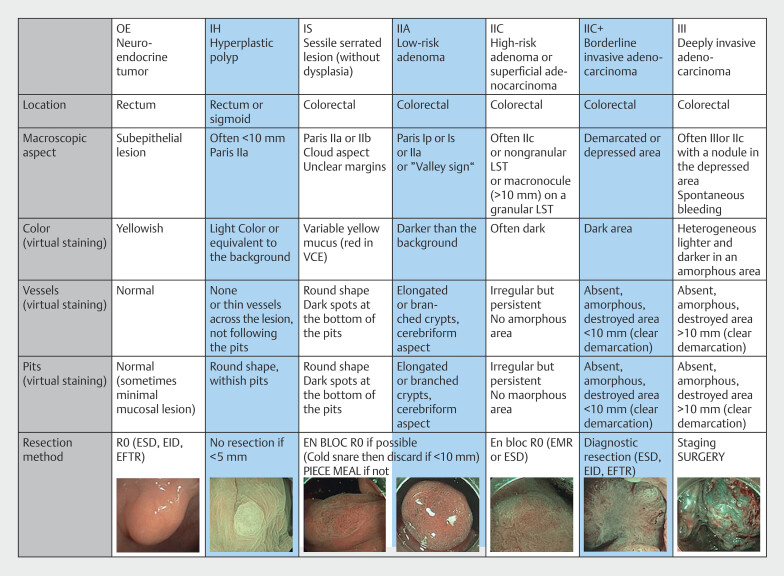
The CONECCT Classification (version 3.1). EID, endoscopic intermuscular dissection; EMR, endoscopic mucosal resection; ESD, endoscopic submucosal dissection; LST, laterally spreading tumor; VCE, virtual chromoendoscopy. Source: Lafeuille, P, Rivory J, Lupu A, et al. Value of green sign and chicken skin aspects for detecting malignancy of colorectal neoplasia in a prospective characterization study. Endoscopy International Open 2024; 12(07); E924-931. doi: 10.1055/a-2350-9631

Colorectal submucosal lesions are lesions that originate beneath the epithelium, in the submucosa, muscularis mucosa, or muscularis propria. They represent a wide variety of cell types, with malignant potential ranging from totally benign to malignant 1 . Optical diagnosis of colorectal lesions is essential to predict histology and choose the most appropriate type of resection. This optical diagnosis fails for submucosal lesions, as the surface is generally normal 2 . Endoscopically visualized submucosal lesions are mainly located in the rectum 3 4 and have recently been described in the CONECCT classification, which proposes advanced resection to achieve R0 resection ( Fig. 1 ). However, submucosal lesions of the colon are much rarer and poorly described, and their common histology is poorly understood. Their resection technique has been little studied, and no clear strategy has been defined. Often, resection techniques for these lesions are similar to those used for rectal submucosal lesions, aiming for R0 en bloc resection, by endoscopic submucosal dissection (ESD) or endoscopic full-thickness resection (EFTR), but without formal proof of efficacy.

Consequently, the question of whether these demanding and not risk-free techniques should be used to treat submucosal lesions of the colon remains uncertain to date and requires further investigation.

Therefore, we conducted a retrospective international multicenter observational study to assess the common histology of colonic submucosal lesions by endoscopic resection or biopsy and determine whether they may always require R0 en bloc endoscopic resection.

## Patients and methods

### Study design

We conducted an international multicenter study based on retrospective data collection of all patients with colonic submucosal lesions with confirmed histology by resection or biopsy between January 2012 and January 2024 in tertiary referral centers in France, Portugal, Italy, and Spain. Selection of the most appropriate strategy for obtaining lesion histology was left to the discretion of the endoscopist at each center.

The ethics committee of Lyon Edouard Herriot Hospital approved this study, and all patients gave informed consent before their procedures. Inclusion criteria were defined as patients of both genders, older than 18 years old; referred to one of the centers for endoscopic resection of a colonic submucosal lesion; and referred to one of the centers for diagnostic or therapeutic colonoscopy, with incidental discovery of a colonic submucosal lesion during examination. The non-inclusion criteria were patients with no colonic submucosal lesion; with colonic submucosal lesion with a typical aspect of lipoma without histological confirmation needed; with a colonic submucosal lesion with non-contributory histological specimen; with a colonic submucosal lesion with a previous attempt of resection; and with a metastatic lesion diagnosed prior to colonoscopy.

### Procedures

All colonoscopies were performed by highly experienced endoscopists, with the patient under general anesthesia and using CO _2_ insufflation. Optical characterization of lesions was performed using high-definition white light endoscopy followed by close-up examination assisted by virtual chromoendoscopy, with or without magnification. Histopathological examination was carried out by expert digestive pathologists.

### Study objectives

The primary endpoint was description of the histology of colonic submucosal lesions.

Secondary endpoints were the description of different lesions in terms of endoscopic aspect and assessment of the proportion of lesions correctly managed by endoscopy, so that the proposed resection technique offered a level of quality of tumor resection adapted to the definitive histology of the lesion, defined by: 1) En bloc R0 resection of malignant lesions using advanced techniques: endoscopic mucosal resection (EMR), EFTR, or ESD; 2) En bloc R0 resection of lesions with local invasive potential; and 3) No resection of non-malignant colonic lesions.

### Data collection

Data collected were patient demographics including sex and age at time of colonoscopy; lesion characteristics: location, size, morphology, and histology; histology confirmation technique (resection or biopsy), residual tumor classification, and adverse events (AEs).

Lesion consistency was defined by resistance when pressed by the endoscope tip or by biopsy forceps. Macroscopic type of lesions was defined as a “hill” aspect when the submucosal curvature showed gentle slopes and as a “sphere” aspect with steep slopes towards the surrounding mucosa.

### Statistical analysis

Continuous variables are presented as mean ± standard deviation. Categorical variables are presented as numbers and percentages.

## Results

### Study population

**Fig. 2:**
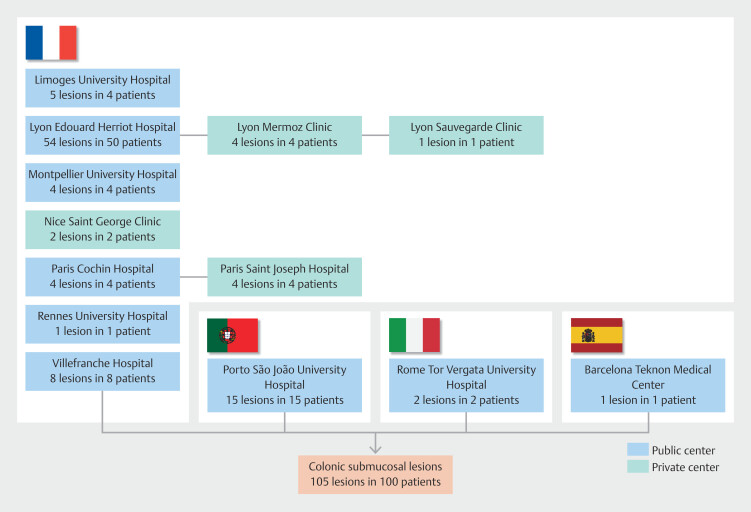
Flow chart of the study.

The study included 100 patients with 105 colonic submucosal lesions from 13 European centers (France: 82 patients, Portugal: 15, Italy: 2, Spain: 1) ( Fig. 2 ). Mean age at diagnosis was 64 years ( Table 1 ). In our tertiary center in Lyon, submucosal lesions accounted for 3.4% of all ESD indications.

**Table:** **Table 1** Characteristics of patients.

Characteristic	
**Patients, n**	100
**Gender, n**	
Male	47
Female	53
**Age at diagnosis, y**	
Mean (SD)	64 (9)
**Center location, n**	
France	82
Portugal	15
Italy	2
Spain	1

### Endoscopic results

Mean lesion size was 13.9 mm. Overall, 58.1% (61/105) of lesions were located beyond the hepatic flexure and 22.9% (24/105) in the sigmoid. Of the lesions, 86.7% (91/105) had the appearance of normal colonic mucosa. Lesion characteristics are detailed in Table 2 .

**Table:** **Table 2** Characteristics of lesions.

Characteristic	
**Lesions, n**	105
**Lesion size: mean (SD), mm**	13.9 (12.3)
**Location, n (%)**	
Appendix	5 (4.8)
Cecum	19 (18.1)
Right colon	31 (29.5)
Hepatic flexure	6 (5.7)
Transverse colon	13 (12.4)
Splenic flexure	0
Left colon	7 (6.7)
Sigmoid	24 (22.9)
**Color, n (%)**	
Buff-yellow	43 (41.0)
Same as background	25 (23.8)
Lighter than background	15 (14.3)
Redish	6 (5.7)
NR	16 (15.2)
**Consistency, n (%)**	
Soft	26 (24.8)
Hard	29 (27.6)
NR	50 (47.6)
**Macroscopic type, n (%)**	
Hill	26 (24.8)
Sphere	26 (24.8)
Pedunculated	15 (14.3)
NR	38 (36.2)
**Mucosal aspect, n (%)**	
Usual colonic	91 (86.7)
Adenoma	1 (1.0)
Scar	3 (2.9)
NR	10 (9.5)
**Histology, n (%)**	
Benign lesions	96 (91.4)
Lipoma	36 (34.3)
Inflammatory lesion	21 (20.0)
Nervous lesion	6 (5.7)
Vascular lesion	5 (4.8)
Leiomyoma	12 (11.4)
Others	16 (15.2)
Malignant lesions	9 (8.6)
GIST	3 (2.9)
Neuroendocrine neoplasia	3 (2.9)
Others	3 (2.9)
**Endoscopy technique, n (%)**	
Forceps	4 (3.8)
Cold snare polypectomy	24 (22.9)
Hot snare polypectomy	1 (1.0)
C-EMR	29 (27.6)
U-EMR	1 (1.0)
C-ESD	27 (25.7)
H-ESD	2 (1.9)
EFTR	17 (16.2)
**Resection, n (%)**	
Biopsy	4 (3.8)
R0	81 (77.1)
R1	20 (19.0)
**Quality, n (%)**	
no resection needed	93 (88.7)
curative	7 (6.7)
not curative	5 (4.8)
**Perforation, n (%)**	
Intraoperative	6 (5.7)
Delayed	0
**Bleeding, n (%)**	
Intraoperative	1 (1.0)
Delayed	0
**Surgery after complication, n (%)**	0
C-EMR, conventional endoscopic mucosal resection; C-ESD, conventional endoscopic submucosal dissection; GIST, gastrointestinal stromal tumor; H-ESD: hybrid endoscopic submucosal dissection; EFTR: endoscopic full-thickness resection; NR, not reported; U-EMR, underwater endoscopic mucosal resection.	

### Histology of lesions

Histology revealed 91.4% (96/105) of non-malignant lesions with 34.3% (36/105) lipomas, 20.0% (21/105) inflammatory lesions, six benign nervous lesions (ganglioneuromas, perineuromas), five benign vascular lesions (angiodysplasias, hemangiomas), 11.4% (12/105) leiomyomas, and 15.2% (16/105) other benign lesions, including two hamartomatous lesions and one desmoid tumor, with local invasive potential. There were 8.6% (9/105) malignant lesions: three gastrointestinal stromal tumors (GISTs), three neuroendocrine neoplasias, one lymphoma, one leiomyosarcoma, and one neuroectodermal tumor.

### Lipomas

Mean size of lipomas was 20.8 mm. Overall, 77.8% (28/36) presented a buff-yellow color. All of the lipomas (18/18) with reported consistency were soft, 47.8% (11/23) of lipomas with reported macroscopic type presented a hill aspect, and 30.4% (7/23) were pedunculated ( Table 3 ).

**Table:** **Table 3** Characteristics of lipomas.

Characteristic	
**Lesions, n**	36
**Lesion size: mean (SD), mm**	20.8 (14.7)
**Location, n (%)**	
Appendix	0
Cecum	10 (27.8)
Right colon	11 (30.6)
Hepatic flexure	2 (5.6)
Transverse colon	3 (8.3)
Splenic flexure	0
Left colon	1 (2.8)
Sigmoid	9 (25)
**Color, n (%)**	
Buff-yellow	28 (77.8)
Same as background	3 (8.3)
Lighter than background	0
Redish	1 (2.8)
NR	4 (11.1)
**Consistency, n (%)**	
Soft	18 (50)
Hard	0
NR	18 (50)
**Macroscopic type, n (%)**	
Hill	11 (30.6)
Sphere	5 (13.9)
Pedunculated	7 (19.4)
NR	13 (36.1)
**Endoscopy technique, n (%)**	
Forceps	4 (11.1)
Cold snare polypectomy	14 (38.9)
Hot snare polypectomy	1 (2.8)
C-EMR	11 (30.6)
U-EMR	0
C-ESD	6 (16.7)
H-ESD	0
EFTR	0
C-EMR, conventional endoscopic mucosal resection; C-ESD, conventional endoscopic submucosal dissection; EFTR: endoscopic full-thickness resection; H-ESD: hybrid endoscopic submucosal dissection; NR, not reported; U-EMR, underwater endoscopic mucosal resection	

### Inflammatory lesions

**Fig. 3:**
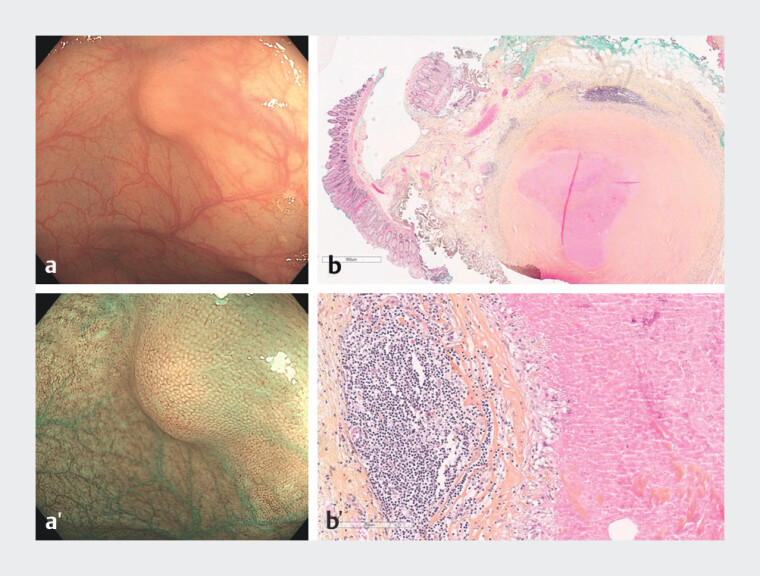
Example of inflammatory submucosal lesion in the colon (granuloma). Endoscopic view in white light ( **a** ) and virtual chromoendoscopy ( **a’** ) and microscopic examination of the resection specimen (HPS staining, low ( **b** ) and high ( **b’** ) magnification).

Mean size of inflammatory lesions was 9.9 mm. Of inflammatory lesions with reported consistency, 92.9% (13/14) were hard and 70.0% (7/10) of inflammatory lesions with reported macroscopic type presented a sphere aspect. Histology revealed 61.9% (13/21) granulomas ( Fig. 3 ), 14.3% (3/21) fibronecrotic granular lesions, and 19.0% (4/21) and 4.8% (1/21) inflammatory granulomatous epithelioid and gigantocellular rearrangements with and without caseous necrosis, respectively ( Table 4 ). Because no patient with caseous necrosis was found to have active tuberculosis, no patient received anti-tuberculosis treatment.

**Table:** **Table 4** Characteristics of inflammatory lesions.

Characteristic	
**Lesions, n**	21
**Lesion size: mean (SD), mm**	9.9 (14.2)
**Location, n (%)**	
Appendix	0
Cecum	5 (23.8)
Right colon	11 (52.4)
Hepatic flexure	1 (4.8)
Transverse colon	2 (9.5)
Splenic flexure	0
Left colon	1 (4.8)
Sigmoid	1 (4.8)
**Color, n (%)**	
Buff-yellow	8 (38.1)
Same as background	6 (28.6)
Lighter than background	3 (14.3)
Redish	1 (4.8)
NR	3 (14.3)
**Consistency, n (%)**	
Soft	1 (4.8)
Hard	13 (61.9)
NR	7 (33.3)
**Macroscopic type, n (%)**	
Hill	2 (9.5)
Sphere	7 (33.3)
Pedunculated	1 (4.8)
NR	11 (52.4)
**Endoscopy technique, n (%)**	
Forceps	0
Cold snare polypectomy	1 (4.8)
Hot snare polypectomy	0
C-EMR	2 (9.5)
U-EMR	0
C-ESD	11 (52.4)
H-ESD	0
EFTR	7 (33.3)
C-EMR: conventional endoscopic mucosal resection; C-ESD: conventional endoscopic submucosal dissection; EFTR: endoscopic full-thickness resection; H-ESD, hybrid endoscopic submucosal dissection; NR, not reported; U-EMR, underwater endoscopic mucosal resection.	

### Endoscopic resections

Endoscopy techniques used included 3.8% (4/105) biopsy forceps and 96.2% (101/105) resections, with 22.9% (24/105) cold snare polypectomies, 0.9% (1/105) hot snare polypectomy, 27.6% (29/105) conventional EMR, 0.9% (1/105) underwater EMR, 25.7% (27/105) conventional ESD, two hybrid ESD, and 16.2% (17/105) EFTR resections.

In addition to the four biopsies, the study included 77.1% (81/105) R0 en bloc resections and 19.0% (20/105) R1 resections. For malignant lesions, en bloc R0 resection was obtained for the four EFTR resections, none (0/1) with conventional ESD resection, and one of the four conventional EMR. For benign lesions requiring en bloc R0 resection, it was obtained for the EFTR resection and the two conventional EMR.

Immediate complications included 5.7% (6/105) perforations and one bleed, all managed endoscopically during the procedure. All perforations occurred after conventional ESD and bleeding occurred after conventional EMR. There were no delayed complications and no need for surgery.

### Appropriateness of endoscopic management

In the study, 11.4% of lesions (12/105) required endoscopic resection and no endoscopic resection was required in 88.7% of cases (93/105). Among the lesions requiring endoscopic resection, 41.7% (5/12) were resected curatively, with en bloc R0 resection of two GISTs in the right colon (conventional EMR and EFTR), a grade 2 neuroendocrine neoplasia in the sigmoid (EFTR), a grade 1 neuroendocrine neoplasia in the left colon (EFTR), a desmoid tumor in the cecum (EFTR), and two hamartomatous lesions in the cecum and appendix (conventional EMR).

In the study, 58.3% (7/12) of lesions requiring endoscopic resection were not resected curatively, with a metastatic grade 2 neuroendocrine neoplasia in the sigmoid (conventional EMR), a neuroectodermal tumor in the left colon (EFTR), a marginal lymphoma in the right colon, with R1 resection (deep margin invasion) by conventional EMR, a leiomyosarcoma in the transverse colon (conventional EMR) and a GIST in the hepatic flexure, with R1 resection (deep margin invasion) by conventional ESD ( Table 5 ).

**Table:** **Table 5** Appropriateness of endoscopic management.

Endoscopic management	
Lesions, n	105
Lesions not requiring endoscopic resection, n (%)	93 (88.7)
Lesions requiring endoscopic resection, n (%)	12 (11.4)
Curative resection, n (%)	5 (41.7)
Not curative resection, n (%)	7 (58.3)

## Discussion

This study demonstrates that submucosal lesions of the colon, although rare, are in a large proportion non-malignant and may not require R0 en bloc endoscopic resection.

In fact, when the lesion resembles a hill with gentle slopes, has a soft consistency and a buff-yellow color, visualization of fat beneath the mucosa after mucosal resection using a cold snare would allow additional biopsy for histological confirmation of a lipoma. This unroofing technique has already been described for large, symptomatic lipomas 5 .

For colonic submucosal lesions without a typical lipoma appearance, spherical in shape and hard in consistency, a low-risk, low-cost histological diagnostic technique such as biopsy forceps or cold snare polypectomy may be sufficient to obtain histology and exclude non-malignant lesions. The effectiveness of simple techniques such as bite-on-bite biopsies or cold snare polypectomy in obtaining histological material of sufficient quality for these hard consistency submucosal lesions, therefore, should be evaluated in the future.

In contrast, when a malignant lesion is diagnosed during the initial sampling, a step-up approach toward advanced endoscopic resection seems justified. A meta-analysis describing efficacy and safety of the EFTR resection technique for colorectal lesions showed an R0 rate and technical success of over 80%, with few adverse effects, but with only 11% (61/555) submucosal lesions 6 . Another meta-analysis showed for ESD of rectal NET a complete resection rate of 89%, 4% adverse events and < 1% local recurrence 7 . In our study, and despite the small number of relevant cases, EFTR also appears to be the most effective technique for achieving en bloc resection with margins, with no reported AEs. Most submucosal lesions were smaller than 20 mm and, consequently, EFTR was almost never contraindicated due to lesion size, because previous reports have shown the relatively low rate of R0 resection beyond 20 mm with this technique 8 . Although ESD is probably better suited to large lesions over 20 mm, the technique seems to have an imperfect R0 resection rate, with a high rate of perforations in this indication, where the depth is difficult to predict and the lesion often buried in the muscle.

The main limitation of our study is due to its retrospective design. First, lesions included were those of undetermined diagnosis, with a probable exclusion of lesions with an obvious lipoma appearance, which could explain the discrepancy between the number of lesions supplied by the centers. Consequently, the proportion of lipomas in the study probably does not reflect the true epidemiology of submucosal colonic lesions. Second, ESD has been used as a means of diagnosis, but the discovered proportion of non-malignant lesions has taught us the need for low-morbidity techniques to obtain histology to decide whether advanced resection is indicated.

## Conclusions

In conclusion, most colonic submucosal lesions are not malignant and, therefore, do not warrant advanced endoscopic resection for R0 purposes. Simple diagnostic techniques for obtaining histology should be tested to propose a step-up selective approach with secondary endoscopic resection (EFTR) for the rare malignant lesions discovered during histological sampling in the first stage.

## References

[1] Standards of Practice Committee Faulx AL Kothari S The role of endoscopy in subepithelial lesions of the GI tract Gastrointest Endosc 2017 85 1117 1132 28385194 10.1016/j.gie.2017.02.022

[2] Veyre F Lambin T Fine C Endoscopic characterization of rectal neuroendocrine tumors with virtual chromoendoscopy: differences between benign and malignant lesions Endoscopy 2021 53 E215 E216 32916731 10.1055/a-1244-9526

[3] Rinke A Ambrosini V Dromain C European Neuroendocrine Tumor Society (ENETS) 2023 guidance paper for colorectal neuroendocrine tumours J Neuroendocrinol 2023 35 e13309 10.1111/jne.13309 37345509

[4] Ramage JK De Herder WW Delle Fave G ENETS Consensus Guidelines Update for Colorectal Neuroendocrine Neoplasms Neuroendocrinology 2016 103 139 143 26730835 10.1159/000443166

[5] Bronswijk M Vandenbroucke A-M Bossuyt P Endoscopic treatment of large symptomatic colon lipomas: A systematic review of efficacy and safety United European Gastroenterol J 2020 8 1147 1154 10.1177/2050640620948661 PMC7724534 32746773

[6] Fahmawi Y Hanjar A Ahmed Y Efficacy and safety of full-thickness resection device (FTRD) for colorectal lesions endoscopic full-thickness resection: A systematic review and meta-analysis J Clin Gastroenterol 2021 55 e27 e36 10.1097/MCG.0000000000001410 33471494 PMC7917149

[7] Zhang H-P Wu W Yang S Endoscopic treatments for rectal neuroendocrine tumors smaller than 16 mm: a meta-analysis Scand J Gastroenterol 2016 51 1345 1353 27367942 10.1080/00365521.2016.1200140

[8] Schmidt A Beyna T Schumacher B Colonoscopic full-thickness resection using an over-the-scope device: a prospective multicentre study in various indications Gut 2018 67 1280 1289 28798042 10.1136/gutjnl-2016-313677

